# Examining social attention as a predictor of problem drinking behavior: A longitudinal study using eye-tracking

**DOI:** 10.1111/acer.15490

**Published:** 2024-12-31

**Authors:** Jiaxu Han, Catharine E. Fairbairn, Walter James Venerable, Sarah Brown-Schmidt, Talia Ariss

**Affiliations:** 1 Department of Psychology, University of Illinois at Urbana-Champaign, Champaign, Illinois, USA; 2 Department of Psychology and Human Development, Vanderbilt University, Nashville, Tennessee, USA

**Keywords:** alcohol, eye-tracking, self-consciousness, social contexts

## Abstract

**Background:**

Researchers have long been interested in identifying objective markers for problem drinking susceptibility informed by the environments in which individuals drink. However, little is known of objective cognitive-behavioral indices relevant to the social contexts in which alcohol is typically consumed. Combining group-based alcohol administration, eye-tracking technology, and longitudinal follow-up over a 2-year span, the current study examined the role of social attention in predicting patterns of problem drinking over time.

**Methods:**

Young heavy drinkers (*N* = 246) were randomly assigned to consume either an alcoholic (target BAC 0.08%) or a control beverage in dyads comprising friends or strangers. Dyads completed a virtual video call in which half of the screen comprised a view of themselves (“self-view”) and half a view of their interaction partner (“other-view”). Participants’ gaze behaviors, operationalized as the proportion of time spent looking at “self-view” and “other-view,” were tracked throughout the call. Problem drinking was assessed at the time of the laboratory visit and then every year subsequent for 2 years.

**Results:**

Significant interactions emerged between beverage condition and social attention in predicting binge drinking days. In cross-sectional analyses, among participants assigned to the control (but not alcohol) group, heightened self-focused attention was linked with increased binge days at baseline, *B* = 0.013, *Exp(B)* = 1.013, 95% CI = [0.004, 0.022], *p* = 0.005. In contrast, longitudinal models indicated that heightened self-focused attention among control participants while interacting with friends was linked with a more pronounced decline in binge drinking over time.

**Conclusions:**

The relationship between social attention and problem drinking is complex and evolves over time. While dispositional self-consciousness may act as a risk factor at the cross-sectional level, it appears to serve a potentially protective function as participants mature into young adulthood. More broadly, results highlight potential utility for objective markers of self-consciousness in the understanding of problem drinking etiology.

## INTRODUCTION

Addiction scientists have long been interested in identifying factors differentiating individuals vulnerable to developing problem drinking (Sayette, 1999). Recently, as direct identification of genetic loci associated with problem drinking has proven difficult (Dick & Foroud, 2003), research programs have been shifting toward extracting endophenotypes more broadly as an alternative approach to the examination of individual differences associated with increased or decreased risk for problem drinking (e.g., Salvatore et al., 2015). A range of endophenotypes for alcohol use disorder (AUD) susceptibility have been explored varying from subjective responses to alcohol (Morean & Corbin, 2010) to event-related potentials (Euser et al., 2012) to serum proteins (Liangpunsakul et al., 2015). More challenging, however, has been the integration of contextual factors into the understanding of mechanisms of risk. The majority of alcohol consumption takes place in the social context (Ally et al., 2016), and social motives represent the most widely endorsed reason for consuming alcohol among young adults (Kuntsche et al., 2005). Recognizing the critical role of social contexts in exacerbating and maintaining problem drinking (McCrady et al., 2006), researchers are increasingly emphasizing expanding the current repertoire of endophenotypes to incorporate indices relevant to the environments drinkers routinely encounter in everyday life (Kendler & Neale, 2010). A key step toward achieving this objective is to extend the study of risk factors of problem drinking beyond intrapersonal to interpersonal research paradigms.

A range of theories has been proposed to explain the role of social processes in alcohol reinforcement and the subsequent development of alcohol problems. Many of the more influential of these theories highlight the role of social-attentional processes in the etiology of AUD (Fairbairn & Sayette, 2014; Hull, 1981; Steele & Josephs, 1990). The “self-awareness model” represents one such theory seeking to elucidate the pathway from environmental and social stressors to problem drinking. The “self-awareness model” proposes that alcohol exerts its rewarding properties by decreasing one’s self-awareness (i.e., self-focused attention) during social interaction (Hull, 1981). Specifically, alcohol is theorized to reduce drinkers’ sensitivity to self-relevant cues regarding appropriate behaviors (Hull, 1981), an effect that may be particularly pronounced and rewarding for people with a natural inclination toward high dispositional levels of self-consciousness in social contexts when sober, as interpersonal interactions tend to prompt self-evaluative processes. Thus, in light of the hypothesized role for alcohol in alleviating an exaggerated and often aversive inward focus, the self-awareness model predicts that individuals with elevated self-focused attention (i.e., high self-consciousness) while not intoxicated are at a greater risk for developing drinking problems (Hull et al., 1986).

Although theory predicts a role for dispositional self-focused attention in the development of problem drinking, results of empirical investigations exploring these associations have been mixed. Regarding cross-sectional findings, whereas some research indicates links between higher levels of self-consciousness and increased alcohol consumption- and alcohol-related problems (Hull et al., 1986; Hull & Young, 1983; LaBrie, Pedersen, et al., 2008), other research indicates the opposite pattern of findings (Chassin et al., 1988; LaBrie, Hummer, & Neighbors, 2008). In longitudinal research, higher levels of self-consciousness have been shown to predict increased drinking among sorority members throughout their college years (Park et al., 2006), increased risk of harm from preloading among university students (Davies & Paltoglou, 2019), as well as increased likelihood of relapse in a clinical sample (Hull et al., 1986). However, several other longitudinal studies have indicated a potentially protective role for self-consciousness in problem drinking risk, including in research exploring underage drinkers (Bartholow et al., 2000; Chassin et al., 1988) and fraternity members (Park et al., 2006). In sum, studies exploring links between self-consciousness and alcohol use have yielded inconsistent findings, including across cross-sectional and longitudinal research paradigms.

A formidable challenge inherent in the study of self-consciousness is the identification of a measure capable of directly assessing social-attentional processes. Despite the demonstrated utility of self-reports in the measurement of many constructs (Robins, 2007), survey-based assessment of self-consciousness raises validity concerns. Specifically, completing any survey measure relevant to oneself inevitably primes the self-schema, thereby inducing increased self-focused attention that can facilitate the retrieval of self-relevant information (Nasby, 1989). Such activation may be harmless in specific assessment contexts, but raises concern surrounding the assessment of self-consciousness, as the subject of the evaluation is being actively triggered during the assessment process itself, and thus the extent to which results of such assessments might generalize to natural (i.e., untriggered) contexts is unknown (Nasby, 1989; Pryor et al., 1977). Of note, prior research exploring links between self-consciousness and drinking patterns has relied predominantly on self-reports, and thus research capable of directly assessing cognitive-behavioral indices of social-attentional processes is implicated.

Attention stands as one of the most extensively studied neurocognitive constructs in psychology and neuroscience research (Carrasco, 2011). Alongside the vast literature produced in examining attention is the maturation of methodologies for its assessment in laboratory settings. Eye-tracking technologies represent one mainstream method for the examination of various cognitive processes, including visual attention (Skaramagkas et al., 2023). Visually guided behavior requires the recruitment of an extensive network of brain regions, thus an assessment of saccadic and gaze behaviors can provide valuable information on the neurocognitive state (Itti, 2015), including social attention (Guillon et al., 2014). Eye-tracking measures have demonstrated adequate internal and test–retest reliability (Ettinger et al., 2003). Importantly, eye-tracking techniques allow for the assessment of gaze behavior in real time within dynamic social interaction, and these methods have thus been employed to measure social attention in virtual social exchange (Ariss et al., 2023; Azriel et al., 2020; Vriends et al., 2017), so yielding objective markers capable of capturing social-attentional processes in an ecologically valid context. Of note, however, most alcohol-administration studies, including those assessing gaze behavior, have relied on noninteractive paradigms (Fairbairn & Sayette, 2014; Maurage et al., 2020, 2021). While research has recently begun integrating methods based on virtual reality, simulating various drinking environments (e.g., bars, parties, driving simulation; Durl et al., 2018; Schneider et al., 2022; Trahan et al., 2019), these platforms have yet to examine unscripted social interaction. Thus, research exploring the effects of alcohol on gaze behavior during dynamic social exchange is implicated.

### The current study

The current study explored links between social attention and problem drinking trajectories over a 2-year span. To the best of our knowledge, the current research represents the first to investigate an objective measure of social-attentional focus as a predictor of problem drinking trajectories observed in real-world contexts. The primary aim of the current study was to examine attentional focus (self vs. other) during social exchange among participants assigned to consume an alcoholic or a control beverage. Leveraging an objective measure of social attention derived from ecologically-valid methods, this work tests predictions derived from self-awareness theory, tracing gaze behavior during dynamic and unscripted virtual social interactions in real time. Were the predictions of the self-awareness model to be borne out, we hypothesized links between higher self-directed attention and problem alcohol use to be moderated by beverage condition, with predictive dispositional tendencies emerging for participants in the control but not alcohol condition (Hull et al., 1986). Specifically, we predicted links between self-focused attention and problem drinking specifically among participants assigned to the control condition, whereas we predicted an attenuation of these associations when self-focused attention was assessed among those assigned to the alcohol condition. As our aim was to understand patterns of problem drinking, the primary outcome measures target variables of binge drinking and negative drinking consequences. Finally, an exploratory aim of the present study was to examine how links might diverge across cross-sectional versus longitudinal models.

## MATERIALS AND METHODS

### Participants

Participants were 246 social drinkers who ranged in age from 21 to 30 (*M* = 22.04, SD = 1.61, 56.1% female) and had 15.44 (SD = 1.49) years of education on average. The sample was predominantly White (64.6%); 23.2% were Asian, 6.9% were Black or African American, 4.1% were Multiracial, and 1.2% were of other ethnicities. Table 1 shows baseline participant demographics and characteristics of drinking behaviors assessed at baseline and longitudinal follow-ups. Participants were recruited via community flyers, online advertisements, and mass email announcements (Ariss et al., 2023). Interested individuals were required to complete a 30-min phone screening to determine eligibility and identify at least one eligible same-gender friend also interested in participating. Individuals were excluded from participation if they (1) had a history of adverse reaction to the amount or type of beverage employed in the study; (2) had a history of alcohol treatment or major problems associated with alcohol, or if they were especially light drinkers or abstainers; (3) had any medical conditions that contraindicate alcohol consumption; (4) dependence on any drug other than nicotine or caffeine; (5) (if female) were pregnant or trying to conceive; (6) had an extreme body mass index (<18 or >35) (also see Ariss et al., 2023).

### Procedure

All procedures employed in the current study were approved by the University of Illinois at Urbana-Champaign Institutional Review Board.

#### Laboratory visit

Eligible participants were invited to the laboratory in pairs, and consent forms were explained and signed upon arrival. Two pairs of friend dyads (i.e., four participants per visit) were typically scheduled to attend the laboratory session on the same day. Such an arrangement allowed for the random assignment of participants to complete experimental procedures either in the company of their own friend (“Friends condition”) or the friend of another participant (“Strangers condition”). To ensure no prior familiarity among participants in the Strangers condition, individuals were casually introduced at the beginning of each visit while their interactions were observed for indications of familiarity (Ariss et al., 2023).

Before beverage administration, participants were asked to provide a breathalyzer reading (Alco-Sensor IV; Intoximeters, St. Louis, Missouri, USA) to ensure that BAC levels were 0.00%. Female participants were asked to also take a urine pregnancy test. Participants’ height and weight were measured to determine beverage dosage. Participants were then brought into separate rooms and completed a battery of questionnaires including those assessing their baseline drinking patterns (see “Measures” section; for a complete list of questionnaires employed in the study, see Ariss et al., 2023). During this time, a light meal adjusted for each participant’s weight was also provided to aid beverage absorption.

##### Beverage administration

After completing baseline questionnaires (e.g., Self-Consciousness Scale; Scheier & Carver, 1985), participants were administered study beverages in assigned pairs. Dyads assigned to the alcohol condition received an alcoholic beverage in the form of a 100-proof vodka and soda mix calculated to induce a peak blood alcohol content (BAC) of 0.08%. The amount of alcohol administered was calculated based on the participant’s age, sex, height, and weight (Watson et al., 1981). Following alcohol administration, participants’ BAC levels were monitored periodically by having them provide a breathalyzer reading every 30 min for the remainder of the visit. Dyads assigned to the control condition received a nonalcoholic soda beverage in an equivalent volume. We did not include a placebo condition (i.e., participants informed that they would be receiving alcohol when in fact they were given a nonalcoholic beverage) in this study as unanticipated compensatory behaviors have emerged in prior studies employing similar placebo manipulations (Testa et al., 2006). All study beverages were divided into three equal portions for administration to ensure an even pacing of consumption. Participants were instructed to drink study beverages over the course of 36 min (every 12 min for each portion), during which time they could interact with each other freely. Immediately post-drink participants engaged in an unrelated electrophysiology task lasting approximately 65 min (Kang et al., 2021). The eye-tracking task—the focus of the current investigation—was timed to coincide with the point at which participants were anticipated to reach peak BAC. A subsample (*N* = 44) of participants completed the eye-tracking task immediately post-drink. Of note, no statistically significant differences were observed for this participant subset in eye-tracking outcomes (Ariss et al., 2023).

##### Eye-tracking task

Dyad members were seated in separate rooms and engaged in two 4-min-long video calls with each other. Participants addressed one of two conversation topics during each video call: music preferences (Topic 1) and likes and dislikes of living in the local community (Topic 2). Participants were informed that the purpose of the video call was to determine the effect of alcohol on visual-response cues during everyday experiences. During each call, one of the two dyad members’ gaze behavior was recorded using a desktop-mounted eye-tracker (EyeLink 1000 Plus; SR Research, Ottawa, Canada) sampled at 1000 Hz. Between conversations, participants switched rooms, so that eye-tracking data were collected from each dyad member once.

For the collection of eye-tracking data, participants placed their heads on a chin rest positioned at a fixed distance from the computer monitor (20-inch screen, 1600 × 1200 resolution). The display of the video call on the monitor was split into two screens of equal sizes, such that each screen occupied exactly half of the monitor. One half displayed the video feed of the participants themselves (“self-view”) while the other half displayed the video feed of their conversation partner (“other-view”). No other content was visible to participants on the screen. Participants were instructed to minimize head movements as much as possible during the call. The side of the screen (left vs. right) assigned to self-view versus other-view was counterbalanced across participants, as was the order of conversation topics (music vs. local community) and the order in which participants were recorded in the task.

After completing the eye-tracking experiment, participants engaged in additional tasks and completed questionnaires unrelated to the current study. Once they finished all tasks for the laboratory session, participants assigned to the control-beverage condition were allowed to leave. Participants who received alcohol were required to stay in the laboratory until their BAC dropped below 0.025%.

#### Longitudinal follow-up

All participants were contacted via email at both 12 (Wave 1) and 24 months (Wave 2) following the date of their initial laboratory visit. Participants were invited to complete an online follow-up survey lasting approximately 20–30 min. To ensure response quality, we incorporated six attention-check/validation items in the surveys (Venerable & Fairbairn, 2020). Responses with three or fewer correct answers to validation items were excluded from further data analysis. Similar to those administered at baseline, the set of questionnaires incorporated in both waves of the follow-up surveys also included questions assessing participants’ problem drinking patterns (see “Measures” section).

### Measures

#### Attention on self versus other during the social exchange

Participants’ attentional focus during the virtual social exchange was assessed using two key variables derived from the eye-tracking task at baseline—proportion fixation on self-view and other-view (Ariss et al., 2023). Note that participants at times fixed their gaze on areas of the screen outside the view comprising the social exchange (e.g., outside the self-view and other-view areas), including on blank portions of the screen. Therefore, although the “self-view” and “other-view” variables were not fully independent, they were also nonredundant (Ariss et al., 2023). Proportion fixation was defined as the amount of time (in milliseconds) each participant spent fixating on a specific screen area (self-view vs. other view) divided by total fixation time for the entire conversation (time spent fixating anywhere on the screen) and then multiplied by a factor of 100. Proportion fixation was chosen as an index well suited to accounting for individual differences in track length and quality (Ariss et al., 2023).

#### Drinking outcomes

Primary outcome measures examined in this research indexed problem drinking behaviors (see preregistration at https://doi.org/10.17605/OSF.IO/DFWKA), defined as patterns of consumption characterized by heavy drinking- and alcohol-related negative consequences (Berkowitz & Perkins, 1986). We incorporated items specifically aimed at measuring binge drinking and adverse drinking consequences. Specifically, *binge drinking days* were assessed by asking participants to report, in the past 30 days, how many days they had five drinks (four drinks if female) or more in one sitting. *Adverse drinking consequences* were assessed via the Short Inventory of Problems scale (SIP-2R; Blanchard et al., 2003). A total of 15 items were included in the SIP-2R, each was rated on a 4-point Likert scale from 0 (Never) to 3 (Daily or almost daily), yielding a total score ranging from 0 to 45. Problem drinking was assessed at baseline as well as at both waves of longitudinal follow-up.

Secondary outcomes assessed overall patterns of alcohol use, including alcohol use frequency (“drinking days”; number of drinking days out of the past 30 days) and quantity (“drinking quantity”; average standard drinks consumed on drinking days over the past 30 days), were also incorporated into both baseline and longitudinal follow-up surveys.

### Data analysis plan

Data and code required for replicating the results of all primary models can be found at https://osf.io/ecwa9/?view_only=1ba76ba0dd0b4edeab06642537efa36f. Main hypotheses and preliminary data analysis procedures were preregistered after data had been collected but before analyses were undertaken; see https://doi.org/10.17605/OSF.IO/DFWKA.

Four participants (1.6%) were excluded due to invalid eye-tracking data (i.e., due to technical difficulties; Ariss et al., 2023). In addition, 17 survey responses were excluded due to failure to demonstrate valid responding via attention-check items (7.3% of Wave 1 responses and 7.9% of Wave 2).

Data analyses were conducted under the framework of multilevel modeling to account for the clustering of drinking behaviors within individuals and also friend dyads. Modeling was conducted at three levels of analysis: within-person (level-1), between-person (level-2), and between-friend dyads (level-3). Primary models predicted problem drinking patterns: binge drinking days and adverse drinking consequences (SIP-2R). In line with the approach taken in our prior work exploring baseline emotion (Ariss et al., 2023), proportion fixations on self-view versus other-view were each entered independently as predictors (see measures section). Time (centered at baseline) was entered at level-1 and the resulting slopes (linear trajectories over time), in addition to intercepts, were treated as random at levels-2 and 3. Beverage condition (alcohol vs. control beverage) was entered at level 3 as a moderator of over-time trajectories in all analyses. Where interactions involved continuous predictors (e.g., proportion self-view or other-view), effects were probed using models centering the continuous predictor at one standard deviation above and below the mean in order to estimate simple contrasts (Cohen & Cohen, 2003).

Visual inspection of binge drinking days and SIP-2R scores suggested each followed a Poisson distribution, and diagnostic procedures indicated no evidence of substantial overdispersion (Kiernan, 2018). Thus, consistent with our prior published studies (Fairbairn & Cranford, 2016; Venerable & Fairbairn, 2020), primary analyses were conducted using multilevel generalized linear models. To permit maximum flexibility in parameter estimates, an unstructured level-1 covariance matrix was employed (Raudenbush & Bryk, 2002). Event Rate Ratios [*Exp(B)*] are reported here as the effect size metric, indicating the percentage change in the dependent variable for each unit increase in the independent variable (Cohen & Cohen, 2003).

## RESULTS

### Sample characteristics and drinking trajectories

Descriptive statistics are presented in Table 1 and correlations between proportion fixation and questionnaire-measured self-consciousness are reported in Table 2. Attrition rates for longitudinal surveys at Wave 1 and Wave 2 were approximately 12% and 20%, respectively. Except for a significant association between responder/nonresponder from Wave 1 and sex (χ^2^(1) = 7.186, *p* = 0.007), where nonresponders (those who did not respond or provided an invalid response) were more likely to identify as male, no other significant differences between responders and nonresponders were observed in age, race, and baseline problem drinking patterns. Correlations among drinking outcomes as assessed at baseline and two waves of longitudinal follow-ups are presented in Supplementary Material (Tables S1).

Consistent with trajectories typically observed in young adulthood, problem drinking behaviors tended to decline over the 2 years of longitudinal follow-up. Specifically, a significant linear decline in binge drinking days was observed, *B* = −0.485, *Exp(B)* = 0.616, 95% Confidence Interval (CI) = [−0.572, −0.397], *t* = −10.87, *p* < 0.0001, with each additional year that passed since the baseline assessment linked with a 38.4% decline in the number of binge drinking days. Adverse drinking consequences (SIP-2R scores) also tended to decline over time, although the decline was not statistically significant, *B* = −0.068, *Exp(B)* = 0.934, 95% CI = [−0.151, 0.015], *t* = −1.61, *p* = 0.109. Similar longitudinal declines were observed for secondary drinking outcomes (drinking days and drinking quantity, see Table S2).

### Cross-sectional associations between gaze fixation and problem drinking

Results indicated a significant interaction between participants’ assigned beverage condition (control vs. alcohol) and self-view fixations in predicting binge drinking days at baseline, *B* = 0.013, *Exp(B)* = 1.013, 95% CI = [0.001, 0.024], *t* = 2.15, *p* = 0.032. Specifically, among participants assigned to receive the control beverage, each 1% increase in time spent fixating on the self-view within the video call corresponded to a 1.3% increase in binge drinking days at baseline, *B* = 0.013, *Exp(B)* = 1.013, 95% CI = [0.004, 0.022], *t* = 2.82, *p* = 0.005. In contrast, among those assigned to receive alcohol, this relationship was nonsignificant, *B* = 0.0002, *Exp(B)* = 1.000, 95% CI = [−0.007, 0.008], *t* = 0.05, *p* = 0.961. Results further indicated a significant interaction between participants’ assigned beverage condition and *other*-view (i.e., conversation partner) fixations in predicting binge drinking days at baseline, *B* = −0.015, *Exp(B)* = 0.985, 95% CI = [−0.026, −0.004], *t* = −2.78, *p* = 0.006 (see “Measures” and “Data analysis plan” sections). Among participants assigned to receive the control beverage, every 1% increase in time spent fixating on the *other*-view within the video call corresponded to a 1.1% decrease in baseline binge days, *B* = −0.012, *Exp(B)* = 0.988, 95% CI = [−0.021, −0.003], *t* = −2.69, *p* = 0.008, whereas this relationship did not reach significance among participants assigned to receive alcohol, *B* = 0.003, *Exp(B)* = 1.003, 95% CI = [−0.003, 0.008], *t* = 0.96, *p* = 0.337. Full model results are presented in Table 3. The interaction between beverage condition and self-/other fixations did not emerge as significant in predicting SIP-2R scores (see Table 3), and no significant interactions with social familiarity (Friends vs. Strangers) emerged at the cross-sectional level (see Table S3).

We next conducted exploratory analyses to examine the extent to which results were driven by *occurrence* (some vs. none) or *frequency* (# within those who displayed any) of binge drinking days among participants. Specifically, the multilevel logistic model suggested that the interaction between beverage condition and self-/other-focused attention did not emerge as significant in predicting the occurrence of binge drinking behaviors (self-view: *B* = −0.034, *t* = −1.01, *p* = 0.311; other-view: *B* = 0.015, *t* = 0.63, *p* = 0.527). Instead, after excluding data points with zero binge drinking days, the multilevel generalized linear model suggested a significant interaction between beverage condition and self-/other-focused attention. Consistent with the above models using the full data-set, increased nonalcohol-induced self-focused attention (and decreased nonalcohol-induced other-focused attention) predicted an increase in the frequency of binge drinking days at baseline (self-view: *B* = 0.012, *t* = 3.15, *p* = 0.002; other-view: *B* = −0.013, *t* = −3.40, *p* < 0.001). The full results of these exploratory analyses were included in the Supplementary Material (Table S4).

Taken together, results of cross-sectional models indicate that, among participants in the control group, increases in self-focused attention correspond to increases in the frequency of binge drinking behavior—associations that disappear among participants in the alcohol group.

### Longitudinal associations between gaze fixation and problem drinking

In longitudinal analyses, the interaction between beverage condition, self-/other-view fixation, and time did not reach significance in predicting either binge drinking days or SIP-2R scores (see Table 3). As an exploratory analysis, we further tested the interaction between beverage condition, self-/other-view fixation, and time in familiar (i.e., Friends) and unfamiliar (i.e., Strangers) contexts, respectively. Whereas the three-way interaction between beverage condition, self-view, and time was significant for those assigned to complete study procedures with a *Friend*, *B* = 0.023, *Exp(B)* = 1.023, 95% CI = [0.007, 0.038], *t* = 2.88, *p* = 0.004, this interaction did not reach significance in the *Stranger* condition, *B* = 0.002, *Exp(B)* = 1.002, 95% CI = [−0.010, 0.014], *t* = 0.30, *p* = 0.762.

To further parse the significant interaction within familiar (i.e., Friend) contexts, we centered self-view fixations at one standard deviation above and below the mean to probe contrasts at each level. Results revealed that the interaction between beverage condition and time emerged as significant for those exhibiting *high self-focus*, *B* = 0.430, *Exp(B)* = 1.537, 95% CI = [0.046, 0.814], *t* = 2.21, *p* = 0.028, but not among those exhibiting *low self-focus*, *B* = −0.333, *Exp(B)* = 0.717, 95% CI = [−0.718, 0.053], *t* = −1.70, *p* = 0.090, during interactions with friends. Individuals who retained a high level of self-focus during interactions with friends after consuming a *control beverage* evinced a marked decline in binge drinking days across the 2 years of longitudinal assessment, amounting to the equivalent of 56.0% a year, *B* = −0.822, *Exp(B)* = 0.440, 95% CI = [−1.114, −0.531], *t* = −5.56, *p* < 0.0001. In contrast, among participants assigned to consume an *alcoholic beverage*, high levels of self-focus were less predictive of over-time trajectories, with the decline in binge drinking behaviors emerging as significantly smaller in this group (32.5%/year), *B* = −0.393, *Exp(B)* = 0.675, 95% CI = [−0.642, −0.143], *t* = −3.09, *p* = 0.002. Full model results are presented in Supplementary Material (Table S5). The three-way interaction did not emerge as significant in predicting SIP-2R scores in either familiar or unfamiliar contexts (see Table S5). Proportion fixation on other-view did not emerge as significant in moderating longitudinal trajectories of problem drinking, either independently (see Table 3) or separately in different social contexts (see Table S5). Taken together, the results of longitudinal models pointed to a complex interplay between individual-level behaviors and context of assessment, with high levels of self-focused attention in familiar social contexts predicting a pronounced decline in binge drinking behaviors over time.

## DISCUSSION

Combining group-based alcohol administration, live tracking of gaze behaviors, and longitudinal follow-up over a 2-year span, the current study was among the first to examine the role of an objective measure of social attention in predicting patterns of problem drinking over time. Overall, results indicated that high levels of self-directed attention among participants assigned to the control condition (but not the alcohol condition) predicted binge drinking in both cross-sectional and longitudinal models. However, both the direction of this effect and also the context for its emergence diverged across longitudinal and cross-sectional analyses. At the cross-sectional level, results indicated that alcohol consumption moderated the relationship between gaze behaviors and binge drinking behaviors such that control-group participants who spent an increased proportion of their time gazing at themselves (and a decreased proportion of time gazing at their conversation partner) reported increased binge drinking frequency. In contrast, a different pattern of results emerged in longitudinal models: control participants who spent a substantially larger proportion of time gazing at themselves during interactions with friends showed a more pronounced decline in binge drinking over time compared to those who spent less time fixating on the self-view. No significant findings emerged in models predicting SIP-2R scores (i.e., adverse drinking consequences). Further, and reinforcing the notion that live/objective and retrospective/subjective measures capture distinct constructs, we observed a nonsignificant correlation between our live gaze-based measures of self-awareness and measures based on reflective self-reports. Results point to a complex role for self-consciousness in problem drinking as captured via a direct measure of social attention, indicating divergent associations across cross-sectional versus longitudinal domains.

Regarding cross-sectional models—indicating links between increased self-focused attention and binge drinking behaviors—findings here are largely in line with the self-awareness model, predicting that the disposition to experience heightened self-consciousness serves as a risk factor that motivates increased drinking (Hull, 1981). These results also extend our prior findings in this sample, in which heightened self-focused attention was associated with increased negative affect following the virtual social exchange (Ariss et al., 2023). Viewed in isolation, the results of these cross-sectional models point to the possibility that individuals with a tendency to direct excessive attention toward themselves in social contexts might turn to drinking as a method of coping with the resulting aversive mental state (Davies & Paltoglou, 2019; Moeller & Crocker, 2009; Pitura & Maranzan, 2018; Skinner & Veilleux, 2016). Of note, however, longitudinal models present an alternative framework for understanding these associations, providing information on alcohol consumption not only at a single moment in time but rather how consumption patterns change and evolve across developmental stages of young adulthood.

Regarding longitudinal models, consistent with a large body of previous research on late adolescents and young adults (Bachman et al., 2014; Bartholow et al., 2000; Lee & Sher, 2018), our study revealed a declining trend in alcohol use across measures of frequency, quantity, and binge days over the course of the study. Data collected from this sample may coincide with a developmental period marked by the “maturing out” phenomenon, characterized by a pattern in which alcohol use peaks in the early twenties and then declines steadily afterward (Arria et al., 2016). Of note, in the current research, the declining trend in binge drinking was particularly prominent among control participants who exhibited heightened self-focused attention during *familiar* social exchange, a finding inconsistent with hypotheses derived from the self-awareness model (Hull et al., 1986). Instead, results appear more in line with research exploring the construct of “protective self-presentational” perspectives within adolescence and young adulthood (Chassin et al., 1988; Crawford & Novak, 2013), pointing to boundary conditions beyond which the self-awareness model does not apply. This body of work suggests that adolescents who are highly self-conscious may be more sensitive to explicit or implicit sanctions against underage drinking within their social environments (e.g., legal system, school, parents, peer norms), thus limiting their alcohol consumption (Chassin et al., 1988). In other words, it is possible that individuals with heightened self-consciousness might also display particular sensitivity and awareness surrounding societal norms and expectations, and thus may ultimately represent a group more likely to monitor and moderate their consumption as development progresses. Regarding the specificity of our longitudinal effects to contexts involving friends, one possibility is that the familiar context served as a more sensitive and (importantly) specific litmus test for identifying those with higher levels of self-consciousness. In particular, while many individuals are likely to experience self-awareness during interactions with strangers (Alden et al., 1992), in contrast only those with particularly elevated dispositional self-consciousness are likely to demonstrate heightened levels of self-focus during interactions with friends.

A variety of factors might explain the divergence in results between cross-sectional versus longitudinal analytic frameworks. First, as with any cross-sectional model, here we are unable to rule out the possibility that the correlation between self-focus and baseline alcohol consumption might reflect one of reverse causality. Just as, in line with predictions of the self-awareness model, it is possible that dispositional self-consciousness might cause excessive drinking, it is also possible that excessive drinking might cause high levels of self-consciousness—a mental state that often exists alongside the depression and anxiety known to result from excessive drinking (Fergusson et al., 2009; Kushner, 2000). Thus, results present the possibility that the cross-sectional associations observed here and also in prior work (Carey, 1995) reflect increased self-focused attention as a consequence, and not a cause, of problem drinking. Alternatively, another framework for understanding divergent results draws on a combined consideration of a maturational/developmental view of alcohol use together with predictions informed by the protective self-presentational perspective (Chassin et al., 1988). Individuals directing more attention to themselves as social object, constantly monitoring how their behavior is viewed by others, might become more sensitive to the explicit or implicit social norms established in drinking contexts (LaBrie, Hummer, & Neighbors, 2008). Thus, they might demonstrate greater sensitivity to environmental and social expectations, evincing a higher level of change as these expectations inevitably shift from heavy drinking norms of the late teens and early twenties to the role transitions and shifting expectations the subsequent years inevitably bring. Indeed, beginning in young adulthood, individuals undergo significant role transitions such as graduating from college, transitioning to employment, partnering up, or embracing independent living (Bachman et al., 2014; Miller-Tutzauer et al., 1991). Research suggested that these role transitions can often result in drastic changes to one’s social environments, contributing to a subsequent decline in heavy drinking (Arria et al., 2016; Lee et al., 2013; Lee & Sher, 2018). Such a mechanistic investigation lies outside the scope of the present work. Future research might directly explore links between changing social expectations across adolescence and young adulthood as these might relate to the role of self-focused attention as an AUD risk.

Of note, although self-focused attention did predict binge drinking behaviors, social-attentional processes measured via eye-tracking were not found to predict adverse drinking consequences in any model. One potential explanation for these findings lies with the specific choice of drinking consequences measure employed in this research. The SIP-2R has proven a reliable indicator of drinking problems (Blanchard et al., 2003). However, the variability in symptomatology captured according to this measure in our sample is relatively low. Although sensitive to more severe alcohol-related problems—for example, physical health harmed by drinking, and severe AUD symptomatology—the SIP-2R scale was originally normed on a population of individuals with AUD and may thus be less sensitive to the level and type of alcohol problems encountered by young adult binge drinkers (Read et al., 2006). Indeed, due to ethical considerations linked with alcohol administration paradigms (National Advisory Council on Alcohol Abuse and Alcoholism, 1989), individuals with a history of severe AUD as well as those with especially light drinking patterns were excluded from this study. This may have also limited the variance captured by drinking outcome variables longitudinally beyond just the SIP-2R. Therefore, it is important to note that findings from the current study may not generalize to populations with a history of more severe forms of AUD. Prior research suggests that high self-consciousness in conjunction with the experience of negative self-relevant events can predict an increased likelihood of relapse following detoxification (Hull et al., 1986). Furthermore, impaired social-cognitive functioning, including heightened self-consciousness, can result from long-term alcohol abuse, thereby compromising the efforts AUD patients make to reduce drinking or maintain abstinence during recovery (Le Berre, 2019). Together, these prior research efforts with clinical samples suggest a different and potentially more nuanced relationship between self-consciousness, problem drinking, and alcohol use intervention than what we were capable of capturing in the context of the current study. Future research is needed to better understand the role of social-attentional processes in predicting drinking and treatment efficacy within the AUD population.

Limitations of the study should be noted. First, we used a virtual platform to assess participants’ attentional processes during social exchange. Virtual contexts reflect an increasingly popular means for engaging in social interaction, especially among younger adults. This method further permits the examination of gaze allocation toward the self and the conversation partner simultaneously. Nonetheless, exploring the application of eye-tracking technology in dynamic in-person social spaces would represent an interesting avenue for future research. Second, our sample consisted primarily of young adults and the longitudinal assessment of their drinking patterns spanned 2 years. Future research might attempt to replicate the study in other age groups as well as extend the span of the longitudinal assessment interval. Further, self-consciousness has been conceptualized as a dispositional construct stable across contexts (Hull, 1981). Eye-tracking measures have demonstrated test–retest reliability in prior research (Ettinger et al., 2003). However, the extent to which the specific measure of social attention employed in the current research might manifest a level of stability consistent with a state-like construct has not been investigated. Third, the current study explored gaze effects at baseline in response to only one alcohol dose, roughly equivalent to a binge drinking episode. Future research might explore the generalizability of effects across variable levels of consumption. Finally, although relatively well powered when compared to other experimental alcohol studies (see Fairbairn et al., 2021; Fairbairn & Sayette, 2014), a larger sample would nonetheless be useful in detecting between-subject interactions that are smaller in magnitude.

In summary, the current study combines eye-tracking technology and longitudinal assessment of alcohol use to examine social-attentional processes as predictors of problem drinking risk. Results indicate that heightened nonalcohol-induced self-focused attention predicted increased binge drinking behavior at baseline yet point to a potential protective role for self-consciousness as participants mature into later stages of young adulthood. This study adds key evidence that social-attentional functioning can capture problem drinking risk, pointing to fruitful avenues of research exploring socially relevant indices in the etiology of problem drinking.

## Supplementary Material

Supplementary Material

## Figures and Tables

**TABLE 1:** Participant demographics and descriptive statistics for drinking outcomes.

Demographics (*N* = 246)			
Age (mean ± SD)			22.04 ± 1.61
Female (*n* (%))			138 (56.1%)
Education years (mean ± SD)			15.44 ± 1.49
Race (*n* (%))			
White			159 (64.6%)
Asian			57 (23.2%)
Black/African American			18 (7.3%)
Multiracial			9 (3.7%)
American Indian/Alaska Native			2 (0.8%)
Native Hawaiian/Other Pacific Islander			1 (0.4%)
Ethnicity (*n* (%))			
Hispanic			44 (17.9%)
Not Hispanic			202 (82.1%)
Beverage condition (*n* (%))			
Alcohol			124 (50.4%)
Control			122 (49.6%)
Familiarity condition (*n* (%))			
Strangers			124 (50.4%)
Friends			122 (49.6%)
Drinking outcomes (mean ± SD [range])			
	Baseline (*N* = 246)	Wave 1 (*N* = 216)	Wave 2 (*N* = 197)
Binge days	4.13 ± 3.64 [0–22]	2.92 ± 3.05 [0–23]	1.64 ± 2.26 [0–10]
Adverse drinking consequences (SIP-2R)	3.30 ± 3.06 [0–17]	3.28 ± 3.75 [0–24]	3.18 ± 3.61 [0–18]
Drinking days	8.47 ± 4.94 [1–28]	7.11 ± 5.55 [0–28]	6.76 ± 5.02 [0–25]
Drinking quantity	4.03 ± 1.95 [1–13]	3.43 ± 2.37 [0–15]	3.01 ± 1.85 [0–10]

Abbreviation: SD, standard deviation.

**TABLE 2:** Pearson correlation matrix of the self-reported, self-consciousness, and proportion fixations measured by eye-tracking (*N* = 122, control condition only).

	1	2	3	4	5
1. Prop. Fix. on self	–				
2. Prop. Fix. on other	−0.886^a^	–			
3. SCS-R private	−0.122	0.113	–		
4. SCS-R public	−0.070	0.120	0.584^a^	–	
5. SCS-R anxiety	−0.110	0.155	0.262^a^	0.409^a^	–

a *p* < 0.01. Prop. Fix. on Self/Other = Proportion of time spent on fixating on the self-/other-view during the virtual social exchange. SCS-R = Self-Consciousness Scale-Revised (Scheier & Carver, 1985).

**TABLE 3:** Alcohol moderates the relationship between proportion fixations and problem drinking at the cross-sectional level.

Dependent variable	Independent variable	*B* (*SE*)	*t*-value	*p*-value
Binge days	Intercept	1.244 (0.102)	12.22	<0.0001
	Ctrl.	−0.125 (0.155)	−0.81	0.421
	Prop. Fix. on Self	0.0002 (0.004)	0.05	0.961
	Ctrl. × Prop. Fix. on Self	0.013 (0.006)	2.15	0.032
	Time	−0.492 (0.079)	−6.24	<0.0001
	Ctrl. × Time	0.025 (0.115)	0.21	0.830
	Prop. Fix. on Self × Time	0.003 (0.003)	1.05	0.292
	Ctrl. × Prop. Fix. on Self × Time	−0.008 (0.005)	−1.67	0.096
Binge days	Intercept	1.052 (0.242)	4.36	<0.0001
	Ctrl.	1.209 (0.430)	2.81	0.006
	Prop. Fix. on Other	0.003 (0.003)	0.96	0.337
	Ctrl. × Prop. Fix. on Other	−0.015 (0.005)	−2.78	0.006
	Time	−0.346 (0.245)	−1.41	0.160
	Ctrl. × Time	−0.456 (0.371)	−1.23	0.220
	Prop. Fix. on Other × Time	−0.001 (0.003)	−0.37	0.712
	Ctrl. × Prop. Fix. on Other × Time	0.004 (0.005)	0.95	0.341
Adverse drinking consequences (SIP-2R)	Intercept	1.043 (0.110)	9.52	<0.0001
	Ctrl.	−0.050 (0.178)	−0.28	0.778
	Prop. Fix. on Self	−0.002 (0.004)	−0.58	0.562
	Ctrl. × Prop. Fix. on Self	0.005 (0.008)	0.62	0.534
	Time	−0.131 (0.080)	−1.65	0.099
	Ctrl. × Time	0.031 (0.132)	0.24	0.813
	Prop. Fix. on Self × Time	0.004 (0.002)	1.51	0.131
	Ctrl. × Prop. Fix. on Self × Time	−0.003 (0.006)	−0.51	0.608
Adverse drinking consequences (SIP-2R)	Intercept	0.766 (0.269)	2.85	0.005
	Ctrl.	0.466 (0.582)	0.80	0.425
	Prop. Fix. on Other	0.003 (0.004)	0.92	0.358
	Ctrl. × Prop. Fix. on Other	−0.006 (0.007)	−0.79	0.432
	Time	0.120 (0.182)	0.66	0.509
	Ctrl. × Time	−0.077 (0.427)	−0.18	0.856
	Prop. Fix. on Other × Time	−0.003 (0.003)	−1.02	0.306
	Ctrl. × Prop. Fix. on Other × Time	0.001 (0.006)	0.15	0.885

*Note*: Ctrl. = a dummy variable with alcohol condition coded as 0 and control condition coded as 1. Prop. Fix. on Self/Other = Proportion of time spent on fixating on the self/other-view during the virtual social exchange. Time = time of assessment with baseline coded as 0, Wave 1 as 1 and Wave 2 as 2. Highlighted rows indicate cross-sectional effects.

## Data Availability

Data and code required for replicating the results of all primary models can be found at https://osf.io/ecwa9/?view_only=1ba76ba0dd0b4edeab06642537efa36f.
