# Supplementary material for: Examining social attention as a predictor of problem drinking behavior: A longitudinal study using eye-tracking
Source: Alcohol Clin Exp Res (Hoboken). Author manuscript; Available in PMC 2025 Jan 18. (PMC11740165; doi:10.1111/acer.15490)
Supplement: Supplementary Material [file NIHMS2046162-supplement-Supplementary_Material.docx]

**Table S1**

*Correlations Among Drinking Outcomes at Baseline, 12- and 24-Month Follow-Up*

|  |  | Drinking Days | | | Drinking Quantity | | | Binge Days | | | Adverse Consequences | | |
| --- | --- | --- | --- | --- | --- | --- | --- | --- | --- | --- | --- | --- | --- |
|  |  | *T0* | *T1* | *T2* | *T0* | *T1* | *T2* | *T0* | *T1* | *T2* | *T0* | *T1* | *T2* |
| Drinking Days | *T0* | 1 | .472^**^ | .481^**^ | -.019 | .090 | .056 | .694^**^ | .326^**^ | .263^**^ | .330^**^ | .297^**^ | .288^**^ |
|  | *T1* | .472^**^ | 1 | .556^**^ | -.080 | .009 | .099 | .269^**^ | .553^**^ | .159^*^ | .109 | .230^**^ | .240^**^ |
|  | *T2* | .481^**^ | .556^**^ | 1 | .017 | .093 | .105 | .337^**^ | .331^**^ | .427^**^ | .175^*^ | .302^**^ | .362^**^ |
| Drinking Quantity | *T0* | -.019 | -.080 | .017 | 1 | .344^**^ | .239^**^ | .384^**^ | .134^*^ | .130 | .218^**^ | .169^*^ | .144^*^ |
|  | *T1* | .090 | .009 | .093 | .344^**^ | 1 | .411^**^ | .252^**^ | .432^**^ | .239^**^ | .117 | .360^**^ | .280^**^ |
|  | *T2* | .056 | .099 | .105 | .239^**^ | .411^**^ | 1 | .158^*^ | .290^**^ | .475^**^ | .143^*^ | .239^**^ | .391^**^ |
| Binge Days | *T0* | .694^**^ | .269^**^ | .337^**^ | .384^**^ | .252^**^ | .158^*^ | 1 | .305^**^ | .284^**^ | .422^**^ | .339^**^ | .332^**^ |
|  | *T1* | .326^**^ | .553^**^ | .331^**^ | .134^*^ | .432^**^ | .290^**^ | .305^**^ | 1 | .242^**^ | .090 | .376^**^ | .343^**^ |
|  | *T2* | .263^**^ | .159^*^ | .427^**^ | .130 | .239^**^ | .475^**^ | .284^**^ | .242^**^ | 1 | .166^*^ | .392^**^ | .513^**^ |
| Adverse  Consequences  (SIP-2R) | *T0* | .330^**^ | .109 | .175^*^ | .218^**^ | .117 | .143^*^ | .422^**^ | .090 | .166^*^ | 1 | .459^**^ | .407^**^ |
|  | *T1* | .297^**^ | .230^**^ | .302^**^ | .169^*^ | .360^**^ | .239^**^ | .339^**^ | .376^**^ | .392^**^ | .459^**^ | 1 | .545^**^ |
|  | *T2* | .288^**^ | .240^**^ | .362^**^ | .144^*^ | .280^**^ | .391^**^ | .332^**^ | .343^**^ | .513^**^ | .407^**^ | .545^**^ | 1 |

*Note.* ^**^. Correlation is significant at the 0.01 level (2-tailed). ^*^. Correlation is significant at the 0.05 level (2-tailed). T0 = Baseline; T1 = 12-month longitudinal follow-up; T2 = 24-month longitudinal follow-up.

**Table S2**

*Overall Trends in Drinking Outcomes over a Two-year Span*

| Dependent Variable | Independent Variable | *B (SE)* | *t*-value | *p*-value |
| --- | --- | --- | --- | --- |
| Binge Days | Time | -0.485 (0.045) | -10.87 | <.0001 |
| Adverse Consequences | Time | -0.068 (0.042) | -1.61 | 0.109 |
| Drinking Days | Time | -0.900 (0.181) | -4.98 | <.0001 |
| Drinking Quantity | Time | -0.482 (0.081) | -5.93 | <.0001 |

*Note*. Time = time of assessment with baseline coded as 0, Wave 1 as 1 and Wave 2 as 2.

**Table S3**

*Social Familiarity and Alcohol Moderate the Longitudinal Relationship between Proportion Fixations on Self-View and Problem Drinking*

| Dependent Variable | Independent Variable | *B* | *SE(B)* | *t*-value | *p*-value |
| --- | --- | --- | --- | --- | --- |
| Binge Days | Intercept | 1.050 | 0.194 | 5.41 | <.0001 |
|  | Time | -0.462 | 0.139 | -3.33 | 0.001 |
|  | Alc. | 0.278 | 0.232 | 1.20 | 0.234 |
|  | Time × Alc. | -0.324 | 0.209 | -1.55 | 0.122 |
|  | Str. | 0.089 | 0.244 | 0.37 | 0.716 |
|  | Time × Str. | 0.031 | 0.168 | 0.19 | 0.853 |
|  | Alc. × Str. | -0.254 | 0.319 | -0.80 | 0.427 |
|  | Time × Alc. × Str. | 0.462 | 0.242 | 1.91 | 0.057 |
|  | Prop. Fix. on Self | 0.020 | 0.010 | 1.89 | 0.060 |
|  | Time × Prop. Fix. on Self | -0.010 | 0.006 | -1.56 | 0.119 |
|  | Alc. × Prop. Fix. on Self | -0.022 | 0.011 | -1.95 | 0.051 |
|  | Time × Alc. × Prop. Fix. on Self | 0.021 | 0.008 | 2.56 | 0.011 |
|  | Str. × Prop. Fix. on Self | -0.010 | 0.011 | -0.87 | 0.382 |
|  | Time × Str. × Prop. Fix. on Self | 0.007 | 0.008 | 0.91 | 0.361 |
|  | Alc. × Str. × Prop. Fix. on Self | 0.016 | 0.014 | 1.13 | 0.261 |
|  | Time × Alc. × Str. × Prop. Fix. on Self | -0.020 | 0.010 | -1.97 | 0.0495 |
| Binge Days | Intercept | 2.505 | 0.722 | 3.47 | 0.001 |
|  | Time | -0.983 | 0.433 | -2.27 | 0.024 |
|  | Alc. | -1.565 | 0.787 | -1.99 | 0.049 |
|  | Time × Alc. | 0.967 | 0.573 | 1.69 | 0.092 |
|  | Str. | -0.409 | 0.804 | -0.51 | 0.612 |
|  | Time × Str. | 0.292 | 0.555 | 0.53 | 0.599 |
|  | Alc. × Str. | 0.602 | 0.940 | 0.64 | 0.523 |
|  | Time × Alc. × Str. | -0.784 | 0.745 | -1.05 | 0.293 |
|  | Prop. Fix. on Other | -0.015 | 0.009 | -1.66 | 0.098 |
|  | Time × Prop. Fix. on Other | 0.005 | 0.006 | 0.86 | 0.393 |
|  | Alc. × Prop. Fix. on Other | 0.020 | 0.010 | 2.02 | 0.044 |
|  | Time × Alc. × Prop. Fix. on Other | -0.012 | 0.007 | -1.65 | 0.101 |
|  | Str. × Prop. Fix. on Other | 0.005 | 0.010 | 0.44 | 0.657 |
|  | Time × Str. × Prop. Fix. on Other | -0.002 | 0.007 | -0.28 | 0.781 |
|  | Alc. × Str. × Prop. Fix. on Other | -0.008 | 0.012 | -0.69 | 0.490 |
|  | Time × Alc. × Str. × Prop. Fix. on Other | 0.012 | 0.009 | 1.26 | 0.207 |
| Adverse Drinking Consequences  (SIP-2R) | Intercept | 1.084 | 0.209 | 5.18 | <.0001 |
|  | Time | -0.043 | 0.132 | -0.33 | 0.744 |
|  | Alc. | 0.121 | 0.267 | 0.45 | 0.650 |
|  | Time × Alc. | -0.221 | 0.193 | -1.15 | 0.252 |
|  | Str. | -0.162 | 0.282 | -0.57 | 0.567 |
|  | Time × Str. | -0.110 | 0.207 | -0.53 | 0.596 |
|  | Alc. × Str. | -0.163 | 0.358 | -0.45 | 0.651 |
|  | Time × Alc. × Str. | 0.354 | 0.265 | 1.34 | 0.182 |
|  | Prop. Fix. on Self | -0.001 | 0.013 | -0.06 | 0.950 |
|  | Time × Prop. Fix. on Self | -0.001 | 0.007 | -0.15 | 0.884 |
|  | Alc. × Prop. Fix. on Self | -0.006 | 0.014 | -0.39 | 0.693 |
|  | Time × Alc. × Prop. Fix. on Self | 0.005 | 0.008 | 0.56 | 0.577 |
|  | Str. × Prop. Fix. on Self | 0.005 | 0.016 | 0.34 | 0.737 |
|  | Time × Str. × Prop. Fix. on Self | 0.003 | 0.010 | 0.32 | 0.746 |
|  | Alc. × Str. × Prop. Fix. on Self | 0.004 | 0.018 | 0.21 | 0.833 |
|  | Time × Alc. × Str. × Prop. Fix. on Self | -0.003 | 0.011 | -0.24 | 0.809 |
| Adverse Drinking Consequences  (SIP-2R) | Intercept | 1.110 | 0.912 | 1.22 | 2.916 |
|  | Time | -0.007 | 0.518 | -0.01 | 1.011 |
|  | Alc. | -0.518 | 0.969 | -0.53 | 1.401 |
|  | Time × Alc. | -0.077 | 0.596 | -0.13 | 1.093 |
|  | Str. | 0.169 | 1.101 | 0.15 | 2.349 |
|  | Time × Str. | 0.095 | 0.740 | 0.13 | 1.549 |
|  | Alc. × Str. | 0.151 | 1.226 | 0.12 | 2.579 |
|  | Time × Alc. × Str. | 0.261 | 0.828 | 0.32 | 1.888 |
|  | Prop. Fix. on Other | 0.000 | 0.011 | -0.04 | 0.022 |
|  | Time × Prop. Fix. on Other | -0.001 | 0.007 | -0.11 | 0.012 |
|  | Alc. × Prop. Fix. on Other | 0.007 | 0.012 | 0.61 | 0.031 |
|  | Time × Alc. × Prop. Fix. on Other | -0.001 | 0.008 | -0.1 | 0.015 |
|  | Str. × Prop. Fix. on Other | -0.003 | 0.014 | -0.23 | 0.024 |
|  | Time × Str. × Prop. Fix. on Other | -0.002 | 0.010 | -0.21 | 0.017 |
|  | Alc. × Str. × Prop. Fix. on Other | -0.003 | 0.016 | -0.22 | 0.027 |
|  | Time × Alc. × Str. × Prop. Fix. on Other | 0.000 | 0.011 | 0.04 | 0.022 |

*Note.* Alc. = a dummy variable with control condition coded as 0 and alcohol condition coded as 1. Str. = a dummy variable with friends condition coded as 0 and strangers condition coded as 1. Prop. Fix. on Self/Other = Proportion of time spent on fixating on the self-/other-view during the virtual social exchange. Time = time of assessment with baseline coded as 0, Wave 1 as 1 and Wave 2 as 2.

**Table S4**

*Alcohol Moderates the Relationship between Proportion Fixations and Problem Drinking at the Cross-sectional Level (Occurrence vs Frequency Model)*

| Dependent Variable | Independent Variable | *B (SE)* | *t*-value | *p*-value |
| --- | --- | --- | --- | --- |
| *Occurrence Model* | | | | |
| Binge Days | Intercept | -3.463 (0.573) | -6.04 | <.0001 |
|  | Ctrl. | 0.574 (0.784) | 0.73 | 0.466 |
|  | Prop. Fix. on Self | 0.010 (0.018) | 0.57 | 0.566 |
|  | Ctrl. × Prop. Fix. on Self | -0.034 (0.033) | -1.01 | 0.311 |
|  | Time | 1.617 (0.365) | 4.43 | <.0001 |
|  | Ctrl. × Time | -0.147 (0.465) | -0.32 | 0.753 |
|  | Prop. Fix. on Self × Time | -0.011 (0.011) | -0.98 | 0.327 |
|  | Ctrl. × Prop. Fix. on Self × Time | 0.019 (0.019) | 1.01 | 0.314 |
| Binge Days | Intercept | -1.677 (1.081) | -1.55 | 0.124 |
|  | Ctrl. | -0.975 (1.768) | -0.55 | 0.582 |
|  | Prop. Fix. on Other | -0.023 (0.017) | -1.40 | 0.162 |
|  | Ctrl. × Prop. Fix. on Other | 0.015 (0.024) | 0.63 | 0.527 |
|  | Time | 0.495 (0.651) | 0.76 | 0.448 |
|  | Ctrl. × Time | 0.598 (0.988) | 0.61 | 0.545 |
|  | Prop. Fix. on Other × Time | 0.013 (0.010) | 1.30 | 0.194 |
|  | Ctrl. × Prop. Fix. on Other × Time | -0.007 (0.014) | -0.49 | 0.622 |
| *Frequency Model* | | | | |
| Binge Days | Intercept | 1.191 (0.098) | 12.19 | <.0001 |
|  | Alc. | 0.096 (0.135) | 0.71 | 0.481 |
|  | Prop. Fix. on Self | 0.012 (0.004) | 3.15 | 0.002 |
|  | Alc. × Prop. Fix. on Self | -0.011 (0.005) | -2.01 | 0.045 |
|  | Time | -0.145 (0.068) | -2.14 | 0.033 |
|  | Alc. × Time | -0.042 (0.104) | -0.41 | 0.685 |
|  | Prop. Fix. on Self × Time | -0.005 (0.003) | -1.86 | 0.064 |
|  | Alc. × Prop. Fix. on Self × Time | 0.006 (0.004) | 1.40 | 0.163 |
| Binge Days | Intercept | 2.396 (0.303) | 7.90 | <.0001 |
|  | Alc. | -1.149 (0.377) | -3.04 | 0.003 |
|  | Prop. Fix. on Other | -0.013 (0.004) | -3.40 | 0.001 |
|  | Alc. × Prop. Fix. on Other | 0.014 (0.005) | 2.97 | 0.003 |
|  | Time | -0.512 (0.186) | -2.75 | 0.006 |
|  | Alc. × Time | 0.274 (0.300) | 0.91 | 0.362 |
|  | Prop. Fix. on Other × Time | 0.004 (0.002) | 1.57 | 0.118 |
|  | Alc. × Prop. Fix. on Other × Time | -0.003 (0.004) | -0.72 | 0.470 |

*Note.* Ctrl. = a dummy variable with alcohol condition coded as 0 and control condition coded as 1. Alc. = a dummy variable with alcohol condition coded as 1 and control condition coded as 0. Prop. Fix. on Self/Other = Proportion of time spent on fixating on the self/other-view during the virtual social exchange. Time = time of assessment with baseline coded as 0, Wave 1 as 1 and Wave 2 as 2. Highlighted rows indicate cross-sectional effects.

**Table S5**

*The 3-way Interaction between Time, Beverage Conditions, and Proportion Fixation in Predicting Drinking under Familiar and Unfamiliar Social Contexts*

| Dependent Variable: Binge Days | | | | Dependent Variable: SIP-2R | | | | |
| --- | --- | --- | --- | --- | --- | --- | --- | --- |
| Independent Variable | *B (SE)* | *t*-value | *p*-value | Independent Variable | *B (SE)* | *t*-value | *p*-value |  |
| *Familiar (Friends) Context* | | | | | | | | |
| Intercept | 1.027 (0.194) | 5.29 | <.0001 | Intercept | 1.087 (0.213) | 5.09 | <.0001 |  |
| Time | -0.437 (0.138) | -3.18 | 0.002 | Time | -0.050 (0.132) | -0.38 | 0.706 |  |
| Alc. | 0.301 (0.230) | 1.31 | 0.196 | Alc. | 0.071 (0.270) | 0.26 | 0.794 |  |
| Time × Alc. | -0.362 (0.203) | -1.79 | 0.075 | Time × Alc. | -0.180 (0.190) | -0.95 | 0.345 |  |
| Prop. Fix. on Self | 0.020 (0.011) | 1.94 | 0.054 | Prop. Fix. on Self | -0.002 (0.013) | -0.17 | 0.863 |  |
| Time × Prop. Fix. on Self | -0.011 (0.006) | -1.77 | 0.078 | Time × Prop. Fix. on Self | 0.001 (0.007) | 0.15 | 0.880 |  |
| Alc. × Prop. Fix. on Self | -0.023 (0.011) | -2.04 | 0.042 | Alc. × Prop. Fix. on Self | -0.002 (0.014) | -0.17 | 0.862 |  |
| Time × Alc. × Prop. Fix. on Self | 0.023 (0.008) | 2.88 | 0.004 | Time × Alc. × Prop. Fix. on Self | 0.002 (0.008) | 0.22 | 0.827 |  |
| Intercept | 2.492 (0.709) | 3.51 | 0.001 | Intercept | 1.106 (0.924) | 1.20 | 0.237 |  |
| Time | -1.005 (0.410) | -2.45 | 0.015 | Time | 0.088 (0.528) | 0.17 | 0.868 |  |
| Alc. | -1.596 (0.774) | -2.06 | 0.044 | Alc. | -0.461 (0.970) | -0.48 | 0.637 |  |
| Time × Alc. | 1.036 (0.542) | 1.91 | 0.057 | Time × Alc. | -0.158 (0.598) | -0.26 | 0.791 |  |
| Prop. Fix. on Other | -0.015 (0.009) | -1.69 | 0.092 | Prop. Fix. on Other | -0.001 (0.011) | -0.06 | 0.951 |  |
| Time × Prop. Fix. on Other | 0.005 (0.005) | 0.98 | 0.327 | Time × Prop. Fix. on Other | -0.002 (0.007) | -0.24 | 0.813 |  |
| Alc. × Prop. Fix. on Other | 0.020 (0.010) | 2.11 | 0.036 | Alc. × Prop. Fix. on Other | 0.007 (0.012) | 0.55 | 0.582 |  |
| Time × Alc. × Prop. Fix. on Other | -0.013 (0.007) | -1.90 | 0.059 | Time × Alc. × Prop. Fix. on Other | 0.000 (0.008) | 0.02 | 0.981 |  |
| *Unfamiliar (Strangers) Context* | | | | | | | | |
| Intercept | 1.189 (0.151) | 7.87 | <.0001 | Intercept | 0.933 (0.183) | 5.09 | <.0001 |  |
| Time | -0.433 (0.094) | -4.59 | <.0001 | Time | -0.179 (0.161) | -1.11 | 0.267 |  |
| Alc. | -0.004 (0.219) | -0.02 | 0.986 | Alc. | -0.046 (0.232) | -0.20 | 0.843 |  |
| Time × Alc. | 0.111 (0.120) | 0.93 | 0.353 | Time × Alc. | 0.141 (0.184) | 0.77 | 0.443 |  |
| Prop. Fix. on Self | 0.007 (0.004) | 1.68 | 0.094 | Prop. Fix. on Self | 0.005 (0.008) | 0.58 | 0.562 |  |
| Time × Prop. Fix. on Self | -0.003 (0.004) | -0.67 | 0.506 | Time × Prop. Fix. on Self | 0.002 (0.007) | 0.31 | 0.755 |  |
| Alc. × Prop. Fix. on Self | -0.005 (0.008) | -0.62 | 0.534 | Alc. × Prop. Fix. on Self | -0.002 (0.011) | -0.19 | 0.852 |  |
| Time × Alc. × Prop. Fix. on Self | 0.002 (0.006) | 0.30 | 0.762 | Time × Alc. × Prop. Fix. on Self | 0.002 (0.008) | 0.26 | 0.799 |  |
| Intercept | 1.963 (0.359) | 5.47 | <.0001 | Intercept | 1.328 (0.585) | 2.27 | 0.027 |  |
| Time | -0.703 (0.332) | -2.12 | 0.035 | Time | 0.076 (0.544) | 0.14 | 0.890 |  |
| Alc. | -0.824 (0.501) | -1.64 | 0.105 | Alc. | -0.451 (0.722) | -0.62 | 0.535 |  |
| Time × Alc. | 0.231 (0.464) | 0.50 | 0.618 | Time × Alc. | 0.180 (0.591) | 0.31 | 0.761 |  |
| Prop. Fix. on Other | -0.009 (0.005) | -1.80 | 0.073 | Prop. Fix. on Other | -0.004 (0.008) | -0.54 | 0.587 |  |
| Time × Prop. Fix. on Other | 0.003 (0.004) | 0.71 | 0.480 | Time × Prop. Fix. on Other | -0.003 (0.007) | -0.41 | 0.683 |  |
| Alc. × Prop. Fix. on Other | 0.010 (0.006) | 1.59 | 0.114 | Alc. × Prop. Fix. on Other | 0.005 (0.010) | 0.52 | 0.603 |  |
| Time × Alc. × Prop. Fix. on Other | -0.001 (0.006) | -0.20 | 0.840 | Time × Alc. × Prop. Fix. on Other | -0.000 (0.008) | -0.02 | 0.980 |  |

*Note.* Alc. = a dummy variable with alcohol condition coded as 1 and control condition coded as 0. Prop. Fix. on Self/Other = Proportion of time spent on fixating on the self/other-view during the virtual social exchange. Time = time of assessment with baseline coded as 0, Wave 1 as 1 and Wave 2 as 2.
